# Supplementary material for: Improvement of islet transplantation by the fusion of islet cells with functional blood vessels
Source: EMBO Mol Med. 2020 Nov 2;13(1):e12616. doi: 10.15252/emmm.202012616 (PMC7799357; doi:10.15252/emmm.202012616)
Supplement: Supplementary file 1 — Appendix [file EMMM-13-e12616-s001.pdf]

Improvement of islet transplantation by the fusion of islet cells with functional blood vessels

Lisa Nalbach[1], Leticia P. Roma[2], Beate M. Schmitt[1], Vivien Becker[1], Christina Körbel[1], Selina Wrublewsky[1], Mandy Pack[1], Thomas Später[1], Wolfgang Metzger[3], Maximilian M. Menger[1,4], Florian S. Frueh[5], Claudia Götz[6], Haopeng Lin[7], Joseline E.M. Fox[7], Patrick E. MacDonald[7], Michael D. Menger[1], Matthias W. Laschke[1], Emmanuel Ampofo[1*]

APPENDIX.

Contents: Appendix Tables S1

Appendix Table S1. Summary of exact *P*-values in figures

Figure		Exact P-value
Fig 2	E	FI vs. CI: P<0.0001; CI vs. PI: P<0.0001; FI vs. PI+MVF: P<0.0001; PI vs. PI+MVF: P<0.0001
	G	FI vs. PI: P=0.015; FI vs. PI+MVF: P<0.0001; CI vs. PI+MVF: P<0.0001; PI vs. PI+MVF: P=0.0002
Fig 3	B	FI vs. CI: P<0.0001; FI vs. PI: P<0.0001; FI vs. PI+MVF: P<0.0001; CI vs. PI: P=0.0004; CI vs. PI+MVF: P<0.0001; PI vs. PI+MVF: P<0.0001
	C	FI vs. CI: P<0.0001; FI vs. PI: P<0.0001; FI vs. PI+MVF: P=0.0012; CI vs. PI+MVF: P<0.0001; PI vs. PI+MVF: P<0.0001
	D	FI vs. PI+MVF: P=0.0009; CI vs. PI+MVF: P=0.0003; PI vs. PI+MVF: P=0.0001
	F	FI 1.1 mM vs. FI 16.5 mM: P=0.003; CI 1.1 mM vs. CI 16.5 mM: P=0.0099; PI 1.1 mM vs. PI 16.5 mM: P=0.0079; PI+MVF 1.1 mM

		vs. PI+MVF 16.5 mM: P=0.002; PI 16.5 mM vs. PI+MVF 16.5 mM: P=0.0487
Fig 4	B	FI vs. CI: P<0.0001; FI vs. PI: P<0.0001; FI vs. PI+MVF: P<0.0001; CI vs. PI+MVF: P<0.0001; PI vs. PI+MVF: P<0.0001
	D	FI vs. PI+MVF: P<0.0001; CI vs. PI+MVF: P<0.0001; PI vs. PI+MVF: P<0.0001
	G	FI vs. PI+MVF: P<0.0001; CI vs. PI+MVF: P<0.0001; PI vs. PI+MVF: P<0.0001
	H	Vehicle vs. Linsitinib: P<0.0001
	J	Vehicle vs. Insulin: P=0.0429
	K	Vehicle vs. Insulin: NS
	M	Day 1:<br>FI vs. PI+MVF: P<0.0001; CI vs. PI+MVF: P<0.0001; PI vs. PI+MVF: P<0.0001<br>Day 2:<br>FI vs. PI+MVF: P<0.0001; CI vs. PI+MVF: P<0.0001; PI vs. PI+MVF: P<0.0001<br>Day 3:<br>FI vs. PI+MVF: P<0.0001; CI vs. PI+MVF: P<0.0001; PI vs. PI+MVF: P<0.0001
Fig 5	D	hFI vs. hPI: P<0.0001; hCI vs. hPI: P<0.0001; hPI vs. hPI+MVF: P<0.0001
	E	hFI vs. hPI: P<0.0001; hFI vs. hPI+MVF: P=0.0002; hCI vs. hPI: P=0.0004; hCI vs. hPI+MVF: P=0.0058
	F	hFI vs. hPI: P<0.0001; hCI vs. hPI: P<0.0001; hCI vs. hPI+MVF: P=0.0021; hPI vs. hPI+MVF: P=0.0152
	G	hFI vs. hPI+MVF: P<0.0001; hCI vs. hPI+MVF: P<0.0001; hPI vs. hPI+MVF: P<0.0001
	I	Day 1:

		hFI vs. hPI+MVF: P<0.0001; hCI vs. hPI+MVF: P<0.0001; hPI vs. hPI+MVF: P<0.0001<br>Day 2:<br>hFI vs. hPI+MVF: P<0.0001; hCI vs. hPI+MVF: P<0.0001; hPI vs. hPI+MVF: P<0.0001<br>Day 3:<br>hFI vs. hPI+MVF: P<0.0001; hCI vs. hPI+MVF: P<0.0001; hPI vs. hPI+MVF: P<0.0001
Fig 6	C	PI vs. PI+MVF: P=0.0266
	E	Day 3:<br>FI vs. PI+MVF: P=0.0024; CI vs. PI+MVF: P<0.0001; PI vs. PI+MVF: P<0.0001<br>Day 6:<br>FI vs. CI: P=0.0351; FI vs. PI: P=0.012; FI vs. PI+MVF: P=0.0017; CI vs. PI+MVF: P<0.0001; PI vs. PI+MVF: P<0.0001<br>Day 10:<br>FI vs. CI: P=0.0133; FI vs. PI: P=0.0162; FI vs. PI+MVF: P=0.0125; CI vs. PI+MVF: P<0.0001; PI vs. PI+MVF: P<0.0001<br>Day 14:<br>FI vs. CI: P=0.0351; FI vs. PI+MVF: P=0.0356; CI vs. PI+MVF: P<0.0001; PI vs. PI+MVF: P<0.0001
	F	Day 3:<br>FI vs. CI: P=0.0352; FI vs. PI: P=0.0244; CI vs. PI+MVF: P=0.0002; PI vs. PI+MVF: P=0.0002<br>Day 6:<br>CI vs. PI+MVF: P=0.0093; PI vs. PI+MVF: P=0.0168<br>Day 10:<br>CI vs. PI+MVF: P=0.0468; PI vs. PI+MVF: P=0.008<br>Day 14:

		FI vs. CI: P=0.0394; FI vs. PI: P=0.0197; CI vs. PI+MVF: P=0.0076; PI vs. PI+MVF: P=0.0035
	H	Day 3:<br>FI vs. PI+MVF: P=0.0032; CI vs. PI+MVF: P<0.0001; PI vs. PI+MVF: P<0.0001<br>Day 6:<br>FI vs. PI+MVF: P<0.0001; CI vs. PI+MVF: P<0.0001; PI vs. PI+MVF: P<0.0001<br>Day 10:<br>FI vs. PI+MVF: P=0.0011; CI vs. PI+MVF: P=0.0001; PI vs. PI+MVF: P=0.0004<br>Day 14:<br>FI vs. PI+MVF: P=0.0002; CI vs. PI+MVF: P<0.0001; PI vs. PI+MVF: P=0.0002
Fig 7	E	FI vs. PI+MVF: P<0.0001; CI vs. PI+MVF: P=0.0005; PI vs. PI+MVF: P<0.0001
Fig 8	D	Day -4:<br>FI vs. Nondiabetic: P<0.0001; PI+MVF vs. Nondiabetic: P<0.0001<br>Day 0:<br>FI vs. Nondiabetic: P<0.0001; PI+MVF vs. Nondiabetic: P<0.0001<br>Day 4:<br>FI vs. PI+MVF: P=0.0006; FI vs. Nondiabetic: P<0.0001; PI+MVF vs. Nondiabetic: P=0.049<br>Day 7:<br>FI vs. PI+MVF: P=0.0051; FI vs. Nondiabetic: P<0.0001<br>Day 10:<br>FI vs. PI+MVF P<0.0001; FI vs. Nondiabetic: P<0.0001

		Day 14:<br>FI vs. PI+MVF P<0.0001; FI vs. Nondiabetic: P<0.0001<br>Day 17:<br>FI vs. PI+MVF P<0.0001; FI vs. Nondiabetic: P<0.0001<br>Day 21:<br>FI vs. PI+MVF P<0.0001; FI vs. Nondiabetic: P<0.0001<br>Day 24:<br>FI vs. PI+MVF P=0.0003; FI vs. Nondiabetic: P<0.0001<br>Day 28:<br>FI vs. PI+MVF P<0.0001; FI vs. Nondiabetic: P<0.0001
	E	FI vs. PI+MVF: P<0.0001; FI vs. Nondiabetic: P<0.0001; PI+MVF vs. Nondiabetic: P<0.0001
	F	45 min: FI vs. Nondiabetic: P=0.0383<br>60 min: FI vs. Nondiabetic: P=0.0267<br>120 min: FI vs. Nondiabetic: P=0.0199<br>180 min: FI vs. Nondiabetic: P=0.022
	G	FI vs. PI+MVF: P=0.0001; FI vs. Nondiabetic: P<0.0001
	K	FI vs. PI+MVF: P<0.0001
Fig EV1	B	FI vs. PI+MVF: P<0.0001; CI vs. PI+MVF: P<0.0001; PI vs. PI+MVF: P<0.0001
	E	FI 1.1 mM vs. FI 16.5 mM: P=0.0002; CI 1.1 mM vs. CI 16.5 mM: P<0.0001; PI 1.1 mM vs. PI 16.5 mM: P=0.0005; PI+MVF 1.1 mM vs. PI+MVF 16.5 mM: P=0.0002
	F	FI vs. CI: P=0.0343; CI vs. PI: P=0.0028; CI vs. PI+MVF: P=0.0052
Fig EV2	D	FI vs. PI+MVF: P=0.0272; PI vs. PI+MVF: P=0.0068
	E	Day 3: 11.0 mM vs. 25.0 mM: P=0.0003
Fig EV3	A	hFI vs. hPI+MVF: P=0.0046; hCI vs. hPI+MVF: P=0.0073
	C	hFI vs. hCI: P=0.0006; hFI vs. hPI: P=0.0004; hFI vs. hPI+MVF:

		P=0.0005
Fig EV4	A	Day 3:<br>FI vs. CI: P=0.026; FI vs. PI+MVF: P=0.0004; CI vs. PI+MVF: P<0.0001; PI vs. PI+MVF: P<0.0001<br>Day 10:<br>FI vs. CI: P=0.007; FI vs. PI: P=0.0411<br>Day 14:<br>FI vs. CI: P=0.0141; CI vs. PI+MVF: P=0.0226
	B	Day 3:<br>FI vs. PI+MVF: P=0.0037; CI vs. PI+MVF: P<0.0001; PI vs. PI+MVF: P<0.0001<br>Day 6:<br>FI vs. PI: P=0.0414
	C	Day 3:<br>CI vs. PI+MVF: P=0.0009; PI vs. PI+MVF: P=0.0012<br>Day 14:<br>FI vs. PI+MVF: P=0.0352
Fig EV5	D	30 min: sham D0 vs. sham D28: P=0.0477<br>60 min: sham D0 vs. sham D28: P=0.0337<br>180 min: sham D0 vs. sham D28: P=0.0403
	E	D0 vs. D28: P=0.0003
	J	FI vs. PI+MVF: P=0.0314
