# Supplementary material for: Improvement of islet transplantation by the fusion of islet cells with functional blood vessels
Source: EMBO Mol Med. 2020 Nov 2;13(1):e12616. doi: 10.15252/emmm.202012616 (PMC7799357; doi:10.15252/emmm.202012616)
Supplement: Supplementary file 2 — Expanded View Figures PDF [file EMMM-13-e12616-s002.pdf]

Expanded View Figures

Figure EV1. Incorporation of MVF into $_{MIN6\beta}$PI, diameters, and insulin secretion of islets and islet organoids.

A Fusion of MIN6β-cells without or with MVF into $_{MIN6\beta}$PI and $_{MIN6\beta}$PI + MVF on day −5, −3 and 0 by means of liquid overlay technique. Scale bar: 150 µm.
B Quantitative analysis of the diameter (µm) of FI, CI, PI, and PI + MVF (n = 15 each). Mean ± SD. One-way ANOVA and Tukey's multiple comparisons *post hoc* test were used for statistical analysis. *$P < 0.05$ vs. FI or hFI; $^{\#}P < 0.05$ vs. CI or hCI; $^{+}P < 0.05$ vs. PI or hPI.
C Neutral red and trypan blue staining of $_{MIN6\beta}$PI, $_{MIN6\beta}$PI + MVF, and $_{MIN6\beta}$PI + MVF incubated for 24 h in 0.2% H_2O_2. Scale bar: 100 µm.
D Length distribution of MVF (%) (n = 3). Mean ± SD.
E Absolute insulin secretion (µU/ml) of FI, CI, PI, and PI + MVF exposed to 1.1 and 16.5 mM glucose (n = 3 each). Mean ± SD. Unpaired Student's *t*-test was used for statistical analysis. *$P < 0.05$ vs. insulin secretion at 1.1 mM glucose within each individual group.
F Insulin SI of FI, CI, PI, and PI + MVF in response to 1.1 and 16.7 mM glucose (n = 3 each). Mean ± SD. One-way ANOVA and Tukey's multiple comparisons *post hoc* test were used for statistical analysis. *$P < 0.05$ vs. FI; $^{\#}P < 0.05$ vs. CI.
G Representative immunofluorescent stainings of insulin and CD31 in $_{MIN6\beta}$PI and $_{MIN6\beta}$PI + MVF. Cell nuclei are stained with Hoechst 33342 (blue). Scale bar: 100 µm.
H Quantitative analysis of insulin- (β-cells) and CD31-positive (endothelial) cells in $_{MIN6\beta}$PI + MVF. Data are expressed in % of all cells (n = 20). Mean ± SD.
I Western blot analysis of CD31 and β-actin in cell extracts of $_{MIN6\beta}$PI, $_{MIN6\beta}$PI + MVF, and MVF.

Data information: Exact *P*-values are specified in Appendix Table S1.

 © 2020 The Authors

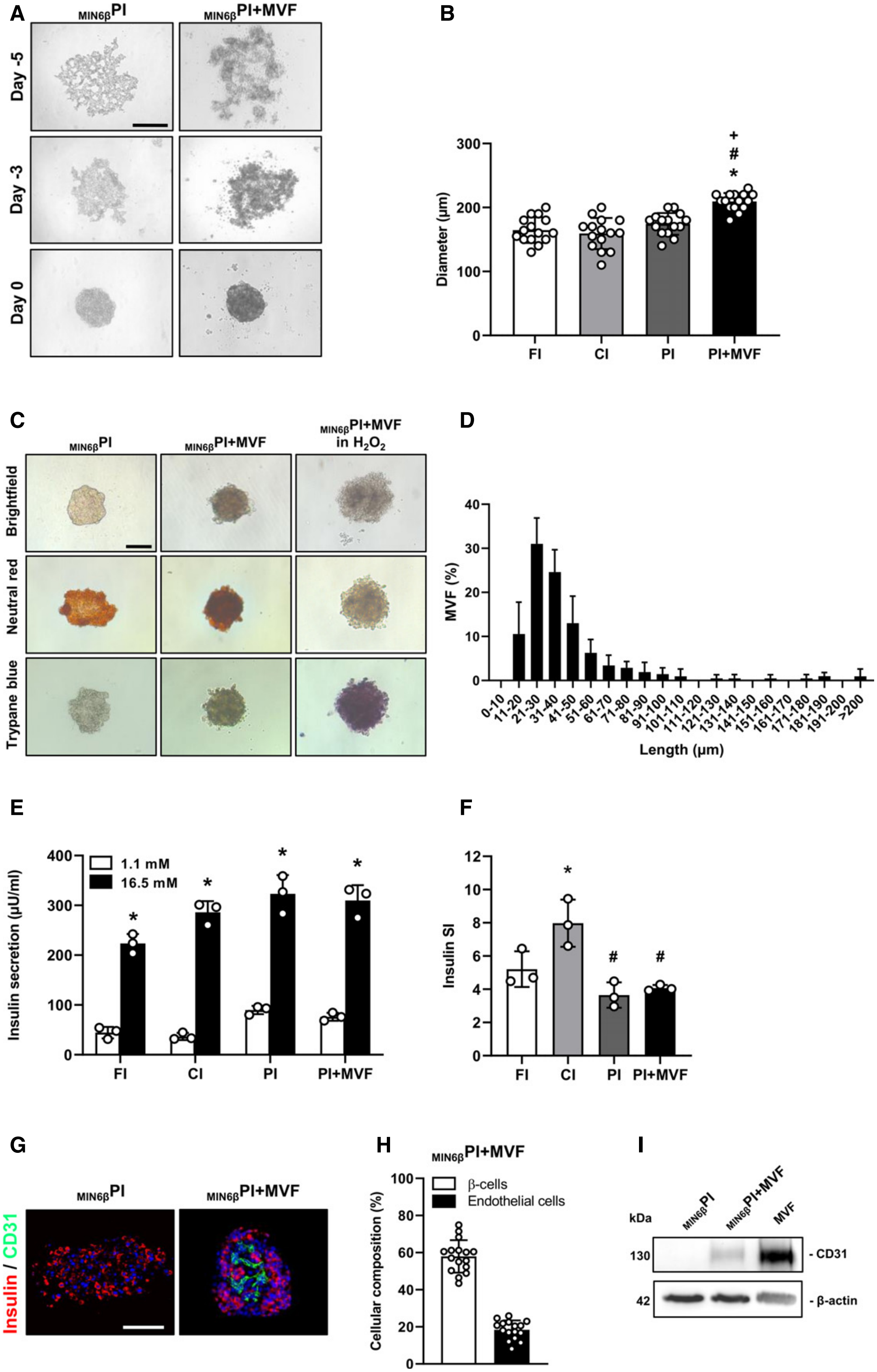
A
MIN6βPI
MIN6βPI+MVF
Day -5
Day -3
Day 0
B
Diameter (µm)
300
200
100
0
FI
CI
PI
PI+MVF
+
#
*
C
MIN6βPI
MIN6βPI+MVF
MIN6βPI+MVF in H_2O_2
Brightfield
Neutral red
Trypane blue
D
MVF (%)
40
30
20
10
0
0-10
11-20
21-30
31-40
41-50
51-60
61-70
71-80
81-90
91-100
101-110
111-120
121-130
131-140
141-150
151-160
161-170
171-180
181-190
191-200
>200
Length (µm)
E
Insulin secretion (µU/ml)
400
300
200
100
0
1.1 mM
16.5 mM
FI
CI
PI
PI+MVF
F
Insulin SI
12
10
8
6
4
2
0
FI
CI
PI
PI+MVF
G
Insulin / CD31
MIN6βPI
MIN6βPI+MVF
H
MIN6βPI+MVF
Cellular composition (%)
100
80
60
40
20
0
β-cells
Endothelial cells
I
kDa
MIN6βPI
MIN6βPI+MVF
MVF
130
42
- CD31
- β-actin

Figure EV1.

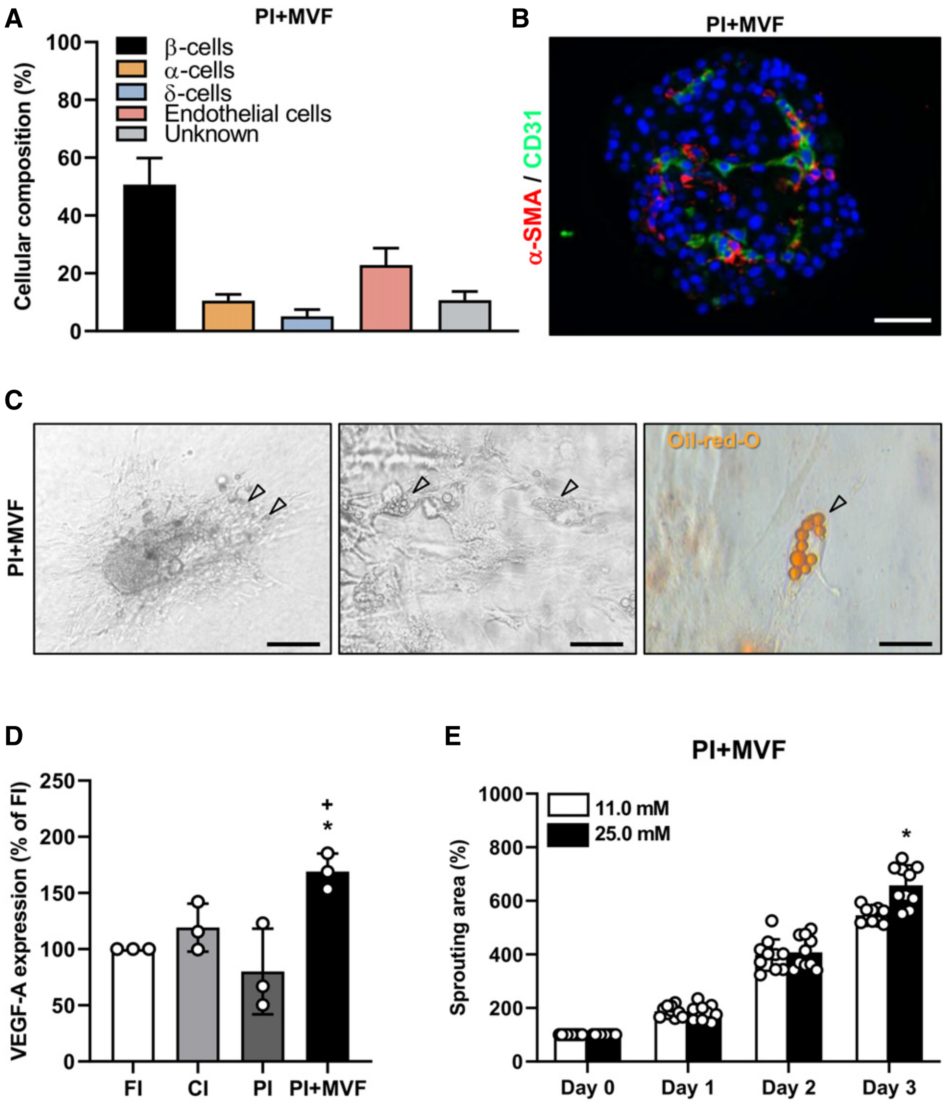

Figure EV2. Cellular composition of PI + MVF, VEGF-A expression, and glucose-dependent sprouting.

A Quantitative analysis of insulin- (β-cells), glucagon- (α-cells), somatostatin- (δ-cells), and CD31-positive (endothelial) cells in PI + MVF. Based on these findings, 11 ± 3% of islet cells (gray bar) are not relatable. Data are expressed in % of total islet cells (*n* = 20). Mean ± SD.

B Representative immunofluorescent staining of α-SMA and CD31 in PI + MVF. Cell nuclei are stained with Hoechst 33342 (blue). Scale bar: 50 μm.

C Bright field images of PI + MVF seeded on culture dish and cultivated for 5 days under 2 μM insulin exposure. Preadipocytes in PI + MVF are marked by arrows. Scale bars: 150 μm (left image) and 30 μm (middle image). Right image shows Oil Red O staining of lipid droplets in preadipocytes of PI + MVF (marked by arrow). Scale bar: 10 μm.

D Quantitative analysis of VEGF-A mRNA-expression in FI, CI, PI, and PI + MVF. Data are expressed as % of FI (*n* = 3 each). Mean ± SD. One-way ANOVA and Tukey's multiple comparisons *post hoc* test were used for statistical analysis. *$P < 0.05$ vs. FI; $^{+}P < 0.05$ vs. PI.

E Quantitative analysis of sprouting areas of PI + MVF exposed to 11.0 mM glucose and 25.0 mM glucose. Data are expressed in % of initial size (day 0; *n* = 10 each). Mean ± SD. Unpaired Student's *t*-test was used for statistical analysis. *$P < 0.05$ vs. 11.0 mM glucose.

Data information: Exact *P*-values are specified in Appendix Table S1.

© 2020 The Authors

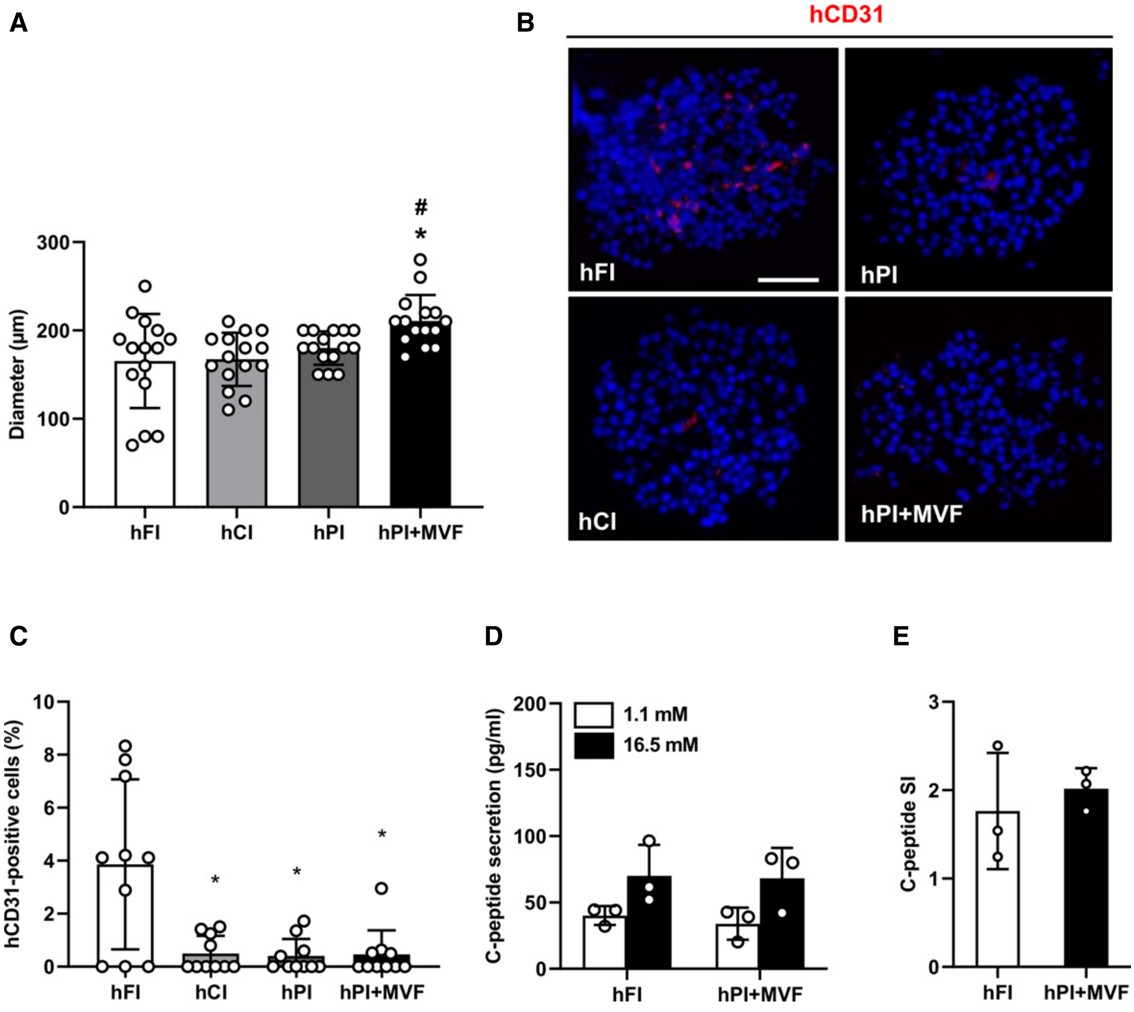

Figure EV3. Diameters, insulin secretion, and CD31 expression of human islets and islet organoids.

A Quantitative analysis of the diameter (µm) of hFI, hCI, hPI, and hPI + MVF (n = 15 each). Mean ± SD. One-way ANOVA and Tukey's multiple comparisons *post hoc* test were used for statistical analysis. $^{*}P < 0.05$ vs. FI or hFI; $^{\#}P < 0.05$ vs. CI or hCI.

B Representative immunofluorescence stainings of human CD31 (hCD31) in hFI, hCI, hPI, and hPI + MVF. Cell nuclei are stained with Hoechst 33342 (blue). Scale bar: 50 µm.

C Quantitative analysis of hCD31-positive cells in hFI, hCI, hPI, and hPI + MVF in % of all islet or organoid cells (n = 10 each). Mean ± SD. One-way ANOVA and Tukey's multiple comparisons *post hoc* test were used for statistical analysis. $^{*}P < 0.05$ vs. hFI.

D Absolute C-peptide secretion (pg/ml) of hFI and hPI + MVF exposed to 1.1 and 16.5 mM glucose (n = 3 each). Mean ± SD.

E C-peptide SI of hFI and hPI + MVF in response to 1.1 and 16.7 mM glucose (n = 3 each). Mean ± SD.

Data information: Exact *P*-values are specified in Appendix Table S1.

© 2020 The Authors

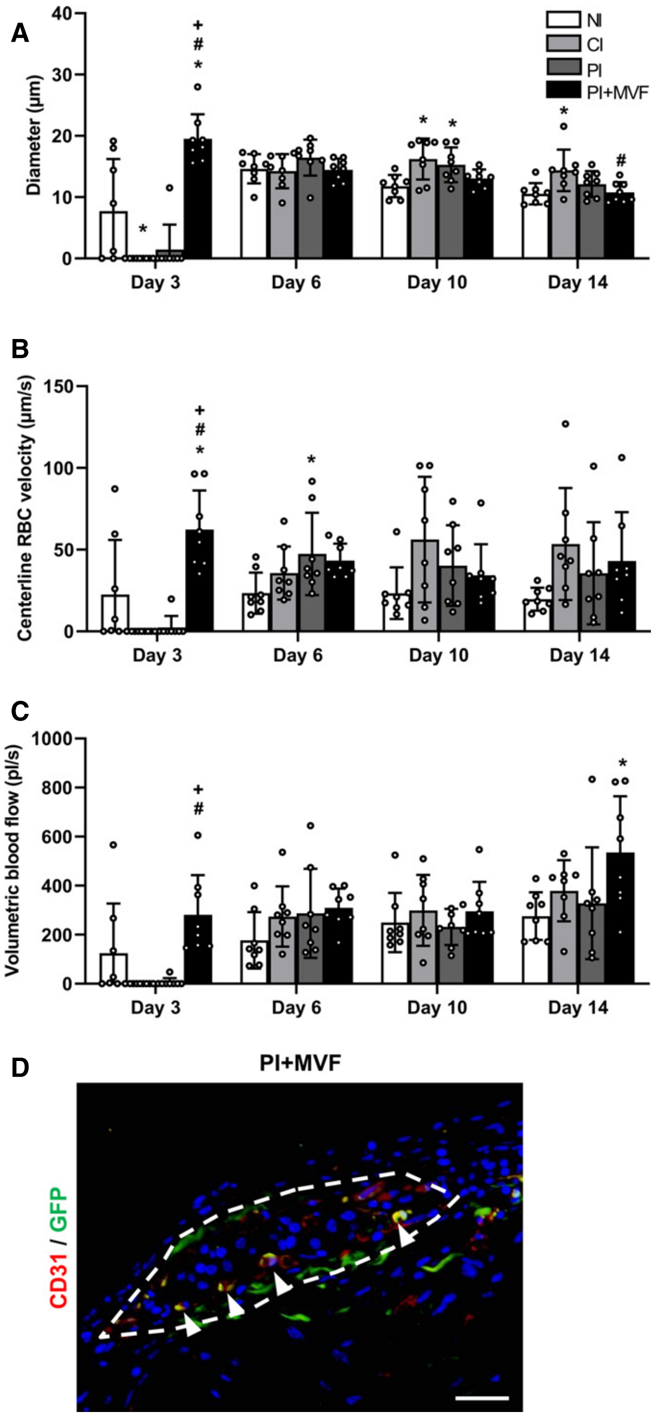

Figure EV4. Microhemodynamic parameters of transplanted FI, CI, PI, and PI + MVF as well as detection of MVF-derived endothelial cells within PI + MVF.

A–C Quantitative analysis of diameter (µm) (A), centerline RBC velocity (µm/s) (B) and volumetric blood flow (pl/s) (C) of newly formed microvessels in transplanted FI, CI, PI, and PI + MVF on day 3, 6, 10, and 14 (n = 8 each). Mean ± SD. One-way ANOVA and Tukey's multiple comparisons *post hoc* test were used for statistical analysis. *$P < 0.05$ vs. FI; $^{\#}P < 0.05$ vs. CI; $^{+}P < 0.05$ vs. PI.

D Representative immunofluorescent stainings of CD31/GFP in PI + MVF consisting of GFP-positive MVF and GFP-negative islet cells on day 14 after transplantation. Cell nuclei are stained with Hoechst 33342 (blue). The border of the PI + MVF is marked by broken line. Arrowheads mark double-positive cells. Scale bar: 40 µm.

Data information: Exact *P*-values are specified in Appendix Table S1.

 © 2020 The Authors

Figure EV5. Identification of the critical islet number necessary for the restoration of normoglycemia in STZ-induced diabetic mice as well as proliferation and apoptosis in FI and PI + MVF after transplantation in diabetic mice.

A Immunofluorescence stainings of insulin in a mouse pancreas treated without and with STZ. Cell nuclei are stained with Hoechst 33342 (blue). Scale bar: 70 μm.
B Quantitative analysis of body weight (g) from day −8 to day 28 of diabetic mice without transplantation (sham group; *n* = 5 each). Mean ± SD.
C Quantitative analysis of blood glucose level (mg/dl) from day −8 to day 28 of animal of the sham group (*n* = 5 each). Mean ± SD.
D Quantitative analysis of blood glucose level (mg/dl) according to the IPGTT of sham-transplanted diabetic mice on day 0 (sham D0) and 28 (sham D28; *n* = 5 each). Mean ± SD. Unpaired Student's *t*-test was used for statistical analysis. *$P < 0.05$ vs. sham D28.
E AUC of IPGTT of sham-transplanted diabetic mice on day 0 (sham D0) and 28 (sham D28; *n* = 5 each). Mean ± SD. Unpaired Student's *t*-test was used for statistical analysis. *$P < 0.05$ vs. sham D28.
F Plasma insulin level (μU/ml) of sham-transplanted diabetic mice on day 0 (sham D0) and 28 (sham D28; *n* = 4 each). Mean ± SD.
G Quantitative analysis of body weight (g) from day −8 to day 28 of diabetic mice transplanted with 200, 250, 300 or 400 FI on day 0 (*n* = 3 each). Mean ± SD.
H Quantitative analysis of blood glucose level (mg/dl) from day −8 to day 28 of diabetic mice transplanted with 200, 250, 300, or 400 FI on day 0 (*n* = 3 each). Mean ± SD.
I Representative stainings of Ki67 and casp-3 in FI and PI + MVF 3 days after transplantation in diabetic mice. Scale bar: 50 μm.
J, K Quantitative analysis of Ki67- (J) and casp-3-positive cells (K) in FI and PI + MVF on day 3 after transplantation in % of all islet or islet organoid cells (*n* = 13 each). Mean ± SD. Unpaired Student's *t*-test was used for statistical analysis. *$P < 0.05$ vs. FI.

Data information: Exact *P*-values are specified in Appendix Table S1.

© 2020 The Authors

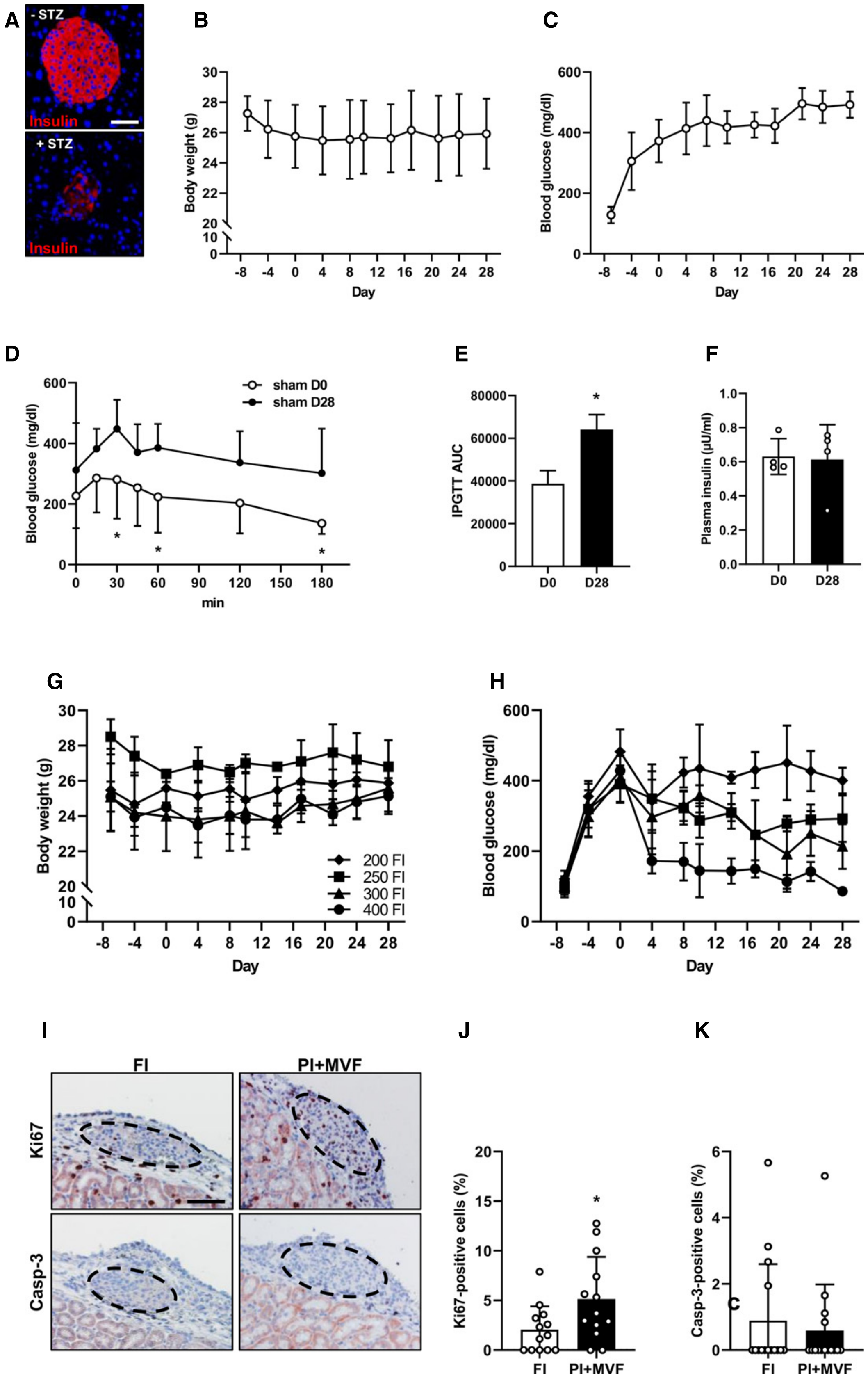
A
- STZ
Insulin
+ STZ
Insulin
B
Body weight (g)
Day
C
Blood glucose (mg/dl)
Day
D
sham D0
sham D28
Blood glucose (mg/dl)
min
E
IPGTT AUC
D0
D28
F
Plasma insulin (µU/ml)
D0
D28
G
Body weight (g)
200 FI
250 FI
300 FI
400 FI
Day
H
Blood glucose (mg/dl)
Day
I
FI
PI+MVF
Ki67
Casp-3
J
Ki67-positive cells (%)
FI
PI+MVF
K
Casp-3-positive cells (%)
FI
PI+MVF

Figure EV5.
