# Supplementary material for: Improvement of islet transplantation by the fusion of islet cells with functional blood vessels
Source: EMBO Mol Med. 2020 Nov 2;13(1):e12616. doi: 10.15252/emmm.202012616 (PMC7799357; doi:10.15252/emmm.202012616)
Supplement: Supplementary file 3 — Table EV1 [file EMMM-13-e12616-s003.docx]

**Table EV1:** Reagents used in the present study.

| **Description** | **Company** |
| --- | --- |
| Agarose | Promega, (Dübendorf, Switzerland) |
| Acrylamide and Bis-acrylamide solution | Bio-Rad Laboratories (München, Germany) |
| Accutase® | BioLegend (Koblenz, Germany) |
| Blotting-Grade Blocker | Bio-Rad Laboratories (München, Germany) |
| Collagenase NB 4G | SERVA GmbH (Heidelberg, Germany) |
| Complete^TM^, Proteaseinhibitor Cocktail | Roche (Basel, Switzerland) |
| Cytofix/Cytoperm^TM^ | Becton Dickinson (San Antonio, USA) |
| DMSO | Fisher Scientific (Schwerte, Germany) |
| DMEM | PAN Biotech (Aidenbach, Germany) |
| ECL Western Blotting Detection Reagent | GE Healthcare GmbH (Freiburg, Germany) |
| Ethanol 100% | Carl Roth GmbH (Karlsruhe, Germany) |
| Ethidiumbromid solution 1% | Carl Roth GmbH (Karlsruhe, Germany) |
| FBS | Biochrom GmbH (Berlin, Germany) |
| FITC-dextran 150.000 | Sigma-Aldrich (Taufkirchen, Germany) |
| Formaldehyd 37% | Merck (Darmstadt, Germany) |
| Glutardialdehyde | Science Services (München, Germany) |
| Glycin | Carl Roth GmbH (Karlsruhe, Germany) |
| HepatoQuick^®^ | Roche (Basel, Switzerland) |
| Hoechst 33342 | Sigma-Aldrich (Taufkirchen, Germany) |
| Isopropanol | Carl Roth GmbH (Karlsruhe, Germany) |
| Neutral red solution | Sigma-Aldrich (Taufkirchen, Germany) |
| Oil Red O | Sigma-Aldrich (Taufkirchen, Germany) |
| PBS | Lonza (Basel, Switzerland) |
| Penicillin | Sigma-Aldrich (Taufkirchen, Germany) |
| Rhodamine 6G | Sigma-Aldrich (Taufkirchen, Germany) |
| SDS | Carl Roth GmbH (Karlsruhe, Germany) |
| Sodiumcacodylate buffer | Carl Roth GmbH (Karlsruhe, Germany) |
| Streptavidin-POD | New England Biolabs (Ipswich, USA) |
| STZ | Sigma-Aldrich (Taufkirchen, Germany) |
| Tween20 | Sigma-Aldrich (Taufkirchen, Germany) |
