# Supplementary material for: Improvement of islet transplantation by the fusion of islet cells with functional blood vessels
Source: EMBO Mol Med. 2020 Nov 2;13(1):e12616. doi: 10.15252/emmm.202012616 (PMC7799357; doi:10.15252/emmm.202012616)
Supplement: Supplementary file 4 — Table EV2 [file EMMM-13-e12616-s004.docx]

**Table EV2:** Antibodies used in the present study.

| **Description** | **Company** |
| --- | --- |
| anti-Akt (4685S) | Cell Signaling (Danvers, USA) |
| anti-BrdU-FITC (11-5071-42) | eBioscience (Waltham, USA) |
| anti-CD31 (DIA310) | Dianova (Hamburg, Germany) |
| anti-CD31 (Sc-4669) | Santa Cruz (Dallas, USA) |
| anti-ERK1/2 (ab115799) | Abcam (Cambridge, UK) |
| anti-GAPDH (sc-25778) | Santa Cruz (Dallas, USA) |
| anti-glucagon (Ab92587) | Abcam (Cambridge, UK) |
| anti-HO-1 (sc-1796) | Santa Cruz (Dallas, USA) |
| anti-insulin (Ab7842) | Abcam (Cambridge, UK) |
| anti-insulin (sc-9168) | Santa Cruz (Dallas, USA) |
| anti-Ki67 (12202) | Cell Signaling (Danvers, USA) |
| anti-mouse IgG (H+L)-Cy3 | Jackson Immuno Research (West grove, USA) |
| anti-pAkt (4060S) | Cell Signaling (Danvers, USA) |
| anti-pERK1/2 (ab50011) | Abcam (Cambridge, UK) |
| anti-rabbit IgG (H+L)-Cy3 | Jackson Immuno Research (West grove, USA) |
| anti-rat IgG (H+L)-Alexa Fluor 555 | Life technologies (Carlsbad, USA) |
| anti-rat IgG (H+L)-Cy3 | Jackson Immuno Research (West grove, USA) |
| anti-somatostatin (Ab30788) | Abcam (Cambridge, UK) |
| anti-αSMA (1A4) | Sigma Aldrich (Taufkirchen, Germany) |
| anti-αSMA (Ab5694) | Abcam (Cambridge, UK) |
| anti-β-Actin (AC-74) | Sigma Aldrich (Taufkirchen, Germany) |
|  |  |
|  |  |
