# Supplementary material for: Improvement of islet transplantation by the fusion of islet cells with functional blood vessels
Source: EMBO Mol Med. 2020 Nov 2;13(1):e12616. doi: 10.15252/emmm.202012616 (PMC7799357; doi:10.15252/emmm.202012616)
Supplement: Supplementary file 5 — Table EV3 [file EMMM-13-e12616-s005.docx]

**Table EV3:** Patient and sample characteristics for the generation of hPI+MVF.

| **Islet isolation ID** | **Donor age (years)** | **Culture time (hours)** | **Shipping media** |
| --- | --- | --- | --- |
| R319 | 69 | 19 | CMRL |
| R321 | 24 | 16 | CMRL |
| R326 | 26 | 35 | CMRL |
| R330 | 39 | 33 | CMRL |
