# Supplementary figures and images for: Improvement of islet transplantation by the fusion of islet cells with functional blood vessels
Source: EMBO Mol Med. 2020 Nov 2;13(1):e12616. doi: 10.15252/emmm.202012616 (PMC7799357; doi:10.15252/emmm.202012616)

## Source data of figure EV1G

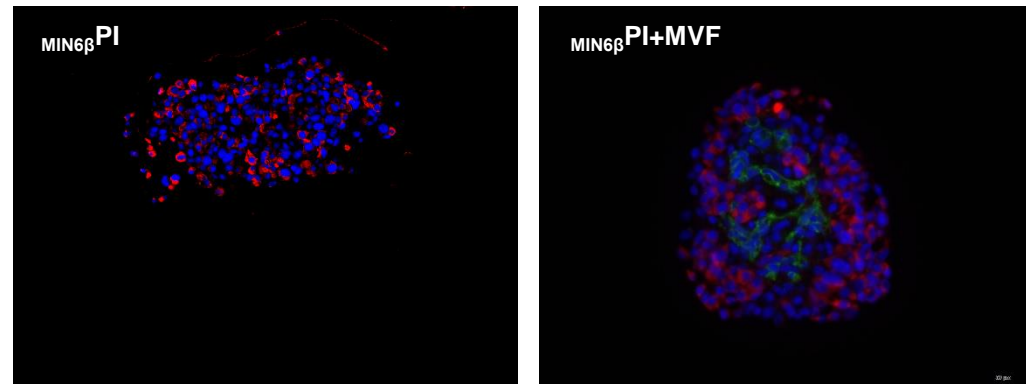

Supplement: Supplementary file 7 — Source Data for Figure 1 [file EMMM-13-e12616-s006.pdf]

## Source data of figure EV3B

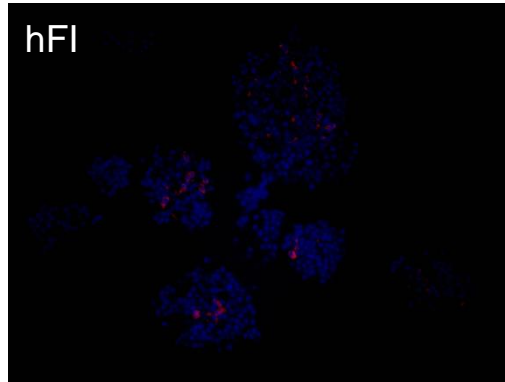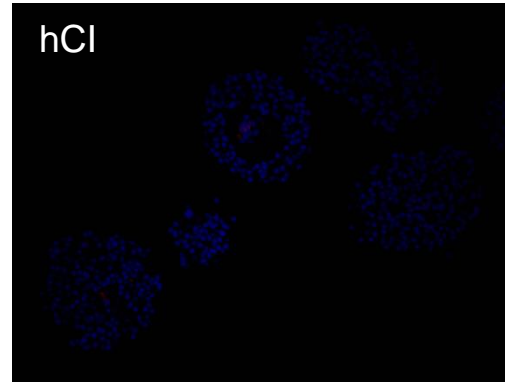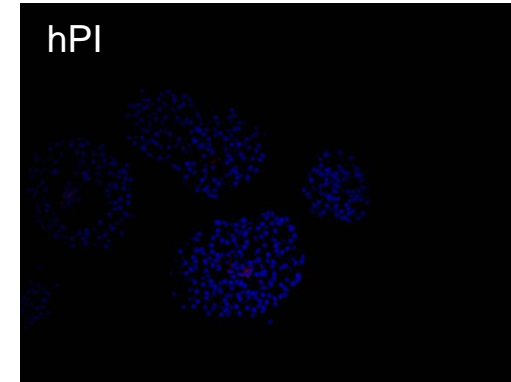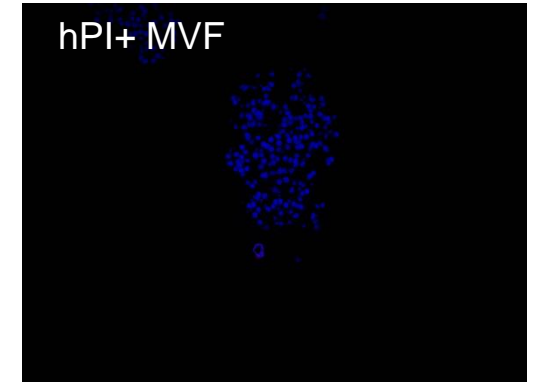

Supplement: Supplementary file 8 — Source Data for Figure 3 [file EMMM-13-e12616-s007.pdf]

# Source data of figure 4A

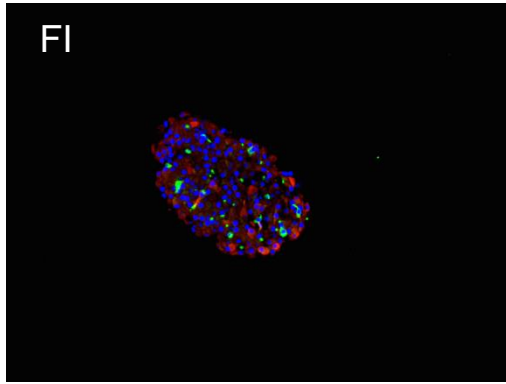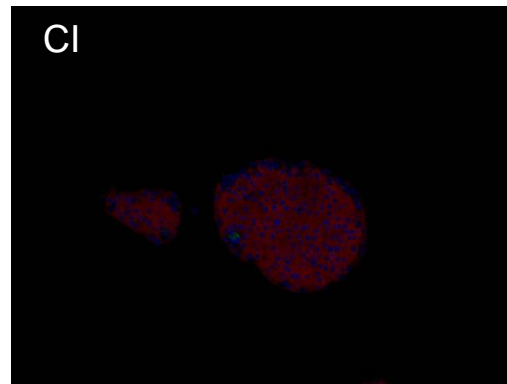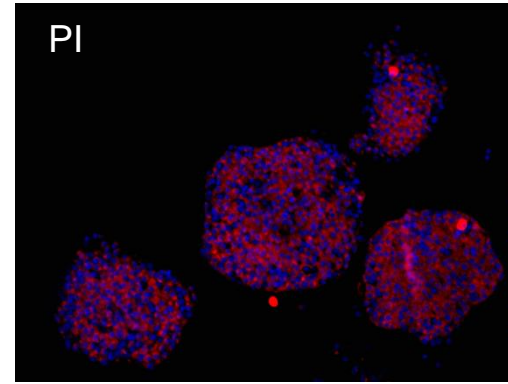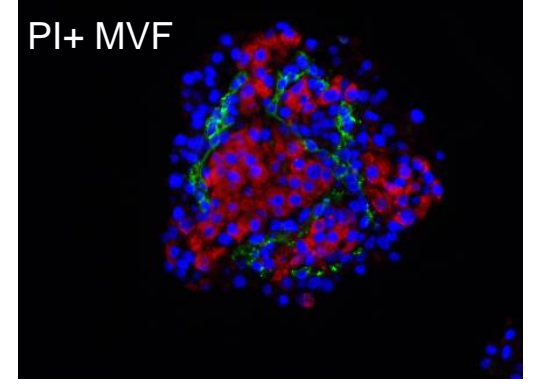

Supplement: Supplementary file 9 — Source Data for Figure 4 [file EMMM-13-e12616-s008.pdf]

## Source data of figure EV5I

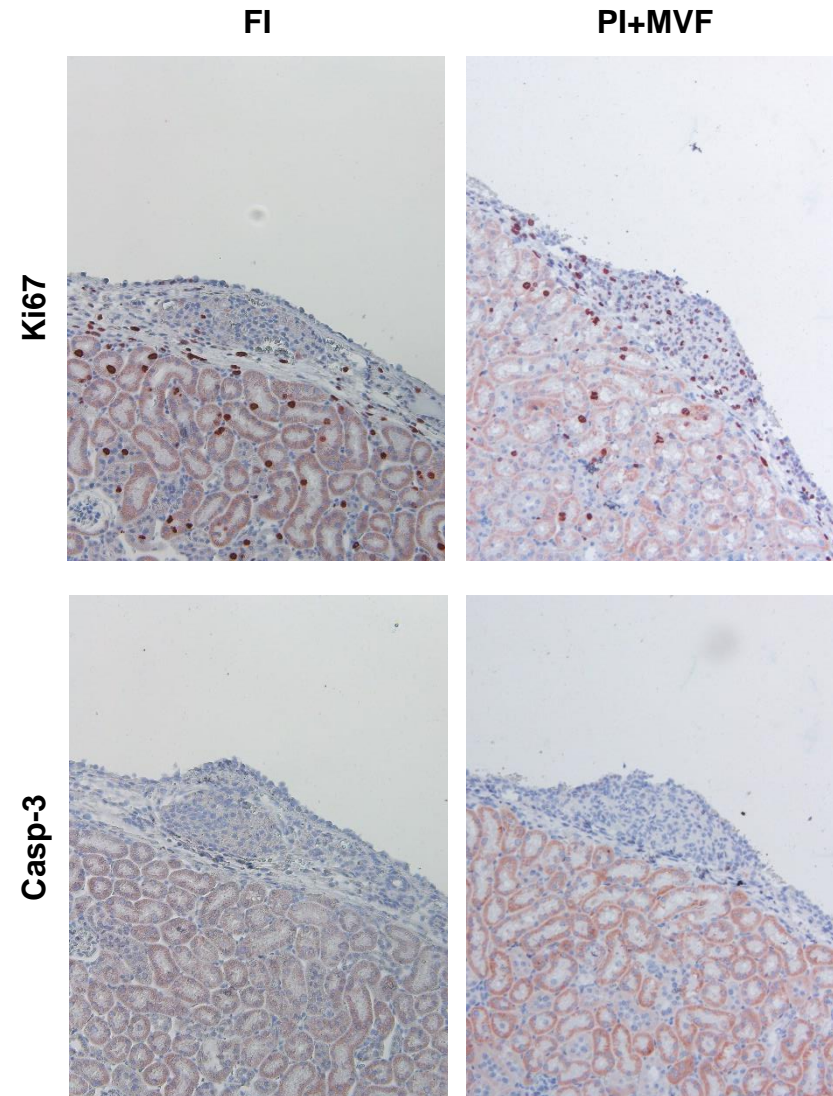

Supplement: Supplementary file 10 — Source Data for Figure 5 [file EMMM-13-e12616-s009.zip › Source_data_Fig_5I.pdf]

## Source data of figure 5C

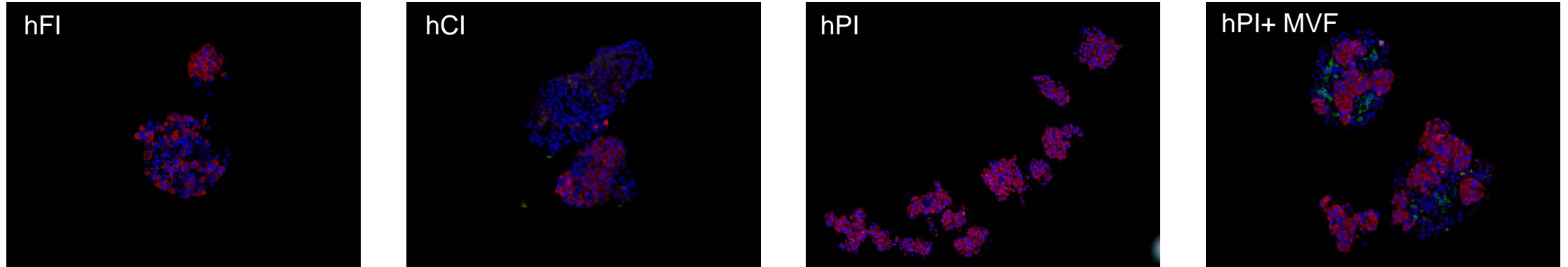

Supplement: Supplementary file 10 — Source Data for Figure 5 [file EMMM-13-e12616-s009.zip › Source_data_Fig_5C.pdf]
